# Functional interaction between orexin/CRF and orexin/dynorphin transmission in the infralimbic cortex modulates the stress-induced reinstatement of alcohol-seeking behavior in male and female Wistar rats

**DOI:** 10.1016/j.neuropharm.2026.110895

**Published:** 2026-02-27

**Authors:** Francisco J. Flores-Ramirez, Rémi Martin-Fardon

**Affiliations:** a Department of Translational Medicine, The Scripps Research Institute, La Jolla, CA, USA; b Department of Psychology, California State University San Marcos, San Marcos, CA, USA

**Keywords:** Alcohol, Orexin, Corticotropin releasing factor, Dynorphin, *Hcrtr1*

## Abstract

Stress contributes to the chronic relapsing nature of alcohol use disorder (AUD), given its ability to elicit craving and precipitate relapse, even after long periods of self-imposed abstinence. Although not thoroughly understood, anatomical, pharmacological, and behavioral data suggest orexin (OX), corticotropin-releasing factor (CRF), and dynorphin (DYN) interact, particularly in the infralimbic cortex (IL). Functional interactions between these three systems in the IL may be critical for the etiology and pervasiveness of compulsive alcohol seeking in dependent subjects, rendering them vulnerable to relapse. Male and female Wistar rats were trained to self-administer 10% alcohol for 3 weeks and then made dependent via chronic intermittent alcohol vapor exposure for 6 weeks. Following extinction training (12 sessions), rats received an intra-IL microinfusion of the OX_1/2_ receptor antagonist suvorexant (15 μg/0.5 μl/side), the CRF_1_ receptor antagonist CP154,526 (0.6 μg/0.5 μl/side), the κ-opioid receptor antagonist nor-binaltorphimine (norBNI; 4 μg/0.5 μl/side), or a combination of suvorexant + CP154,526 or suvorexant + norBNI. Rats were then tested for footshock stress-induced reinstatement of alcohol-seeking behavior. In nondependent rats, only CP154,526 prevented the reinstatement of alcohol-seeking behavior, an effect that was reversed by suvorexant co-administration. In dependent rats, suvorexant, CP154,526, and norBNI attenuated alcohol-seeking behavior. Interestingly, the co-administration of suvorexant + CP154,526 or suvorexant + norBNI attenuated suvorexant’s effect. Increases in *Hcrtr1* mRNA expression in the IL were found in alcohol-dependent rats only. These results demonstrate a functional interaction between OX/CRF and OX/DYN receptor signaling in the IL in subjects with a history of alcohol dependence.

## 1. Introduction

Corticotropin-releasing factor (CRF) regulates responses to stress across physiological systems, including immune, endocrine, and neural pathways (Vale et al., 1981). Several neuropeptides that been suggested to interact with CRF to influence drug-seeking behavior, including relapse to alcohol use (Shalev et al., 2010). The orexin (OX) system is implicated in motivational effects of addictive substances, is activated by stress, and may modulate responses to stress (Bonci and Borgland, 2009; Giardino and de Lecea, 2014; Harris et al., 2005; Ida et al., 2000; Thompson and Borgland, 2011). Anatomically, CRF neurons from the prefrontal cortex synapse with hypothalamic OX cells that express CRF receptors, and reciprocal connections exist between OX and CRF pathways (Winsky-Sommerer et al., 2004). Behaviorally, OX administration increases anxiety-like behavior and reinstates drug seeking in a CRF-dependent manner, indicated by elevations of intracranial self-stimulation thresholds and an increase in cocaine-seeking behavior in preclinical animal models (Boutrel et al., 2005; Hata et al., 2011; Suzuki et al., 2005). Altogether, these findings indicate that OX-CRF interactions contribute to persistent negative emotional states that drive dependence.

The dynorphin (DYN)/κ-opioid receptor (KOP) system is widely distributed in the central nervous system (Watson et al., 1982) and plays a key role in the development of behavioral alterations that are consistent with the “dark side” of AUD (Koob, 2015). Interestingly, the majority (94%) of hypothalamic neurons that express *Ox* also co-express prodynorphin (*Pdyn*) mRNA, suggesting a functional overlap (Chou et al., 2001; Li & van den Pol, 2006). Orexin and DYN are co-packaged and released from synaptic vesicles, which may enable their interaction in brain regions that underlie the exacerbation of Drugs-seeking behavior (Li & van den Pol, 2006; Muschamp et al., 2014). Notably, although both are released by electrical stimulation of the hypothalamus (HYP), they play opposing roles in cocaine self-administration, brain stimulation reward, impulsivity, and ventral tegmental area (VTA) neuron firing rate (Li & van den Pol, 2006; Muschamp et al., 2014). Notably, when co-applied, no net changes in VTA dopamine neuron firing were found in mice, suggesting balanced and opposing effects of OX and DYN (Muschamp et al., 2014). Specifically, in mice, blocking OX1 receptors in the VTA elevated thresholds for brain stimulation reward, an effect that was prevented by KOP antagonism, indicating an OX-DYN interplay in reward modulation (Muschamp et al., 2014). Similarly, in rats with prolonged access to cocaine, OX administration in the paraventricular nucleus of the thalamus (PVT) reinstated drug seeking, but co-administration with DYN blocked this effect by reducing glutamate release. Notably, this interaction did not affect OX-driven palatable food seeking, suggesting alterations of OX-DYN dynamics by drug dependence in reward pathways (Matzeu et al., 2018).

The medial prefrontal cortex (mPFC) is a central component of the affective cortico-limbic-striatal system that regulates behavior that is motivated by both reward and aversion (Peters et al., 2009). Consisting of the dorsal region (i.e., prelimbic cortex [PL]) and ventral region (i.e., infralimbic cortex [IL]), which have largely distinct projection patterns to the nucleus accumbens, amygdala, and HYP (Heidbreder and Groenewegen, 2003; Vertes, 2004, 2006), a functional dichotomy has been described in rodents. The PL and IL both receive robust projections from the ventral hippocampus (Hoover and Vertes, 2007; Jin and Maren, 2015; Kim and Cho, 2017), which is implicated in the processing of appetitive and aversive contextual information (Good and Honey, 1991; Mankin et al., 2015; Maren and Holt, 2000; Penick and Solomon, 1991; Riaz et al., 2017; Sutherland and McDonald, 1990; Sutherland et al., 1989; Wagatsuma et al., 2018). The PL and IL monitor changing environmental contexts to orchestrate the most contextually appropriate behavior through their efferent projection sites. Importantly, the mPFC has been implicated in the context-induced reinstatement of drug seeking (Bossert et al., 2011; Di Pietro, Black and Kantak, 2006) and contextual fear expression (Corcoran and Quirk, 2007). The PL plays a role in executing behaviors, while the IL is involved in response inhibition (Moorman and Aston-Jones, 2015; Moorman et al., 2015). Early studies suggested that the PL drives (promotes) drug seeking, whereas the IL suppresses it (e.g., IL activation was associated with the extinction and inhibition of drug-seeking behavior; for review, see (Van den Oever et al., 2010). This strict functional dichotomy of PL-Go/IL-Stop circuitry was recently shown to depend on the specific behavioral models that are used, and the IL was suggested to play a role in both the promotion and inhibition of drug (and food) seeking (Muller Ewald et al., 2019; Warren et al., 2019). Alcohol was shown to affect mPFC functionality (Holmes et al., 2012; Hu et al., 2015; Kroener et al., 2012; Pava and Woodward, 2014), with more robust changes in the IL *vs.* PL (Pleil et al., 2015). Alcohol exposure induced hyperexcitability of the IL in mice (Pleil et al., 2015), and a prolonged history of alcohol dependence caused substantial and long-lasting reorganization of the IL. Ultimately, evidence that implicates the IL in the inhibition of excessive alcohol seeking was described using the Daun02 technique, in which neuronal ensemble inactivation in the IL but not PL resulted in excessive alcohol seeking (Pfarr et al., 2015), suggesting that IL dysregulation may be a major contributor to the loss of control of alcohol seeking in AUD.

Direct experimental evidence is lacking about how IL neurons respond to the simultaneous release of OX and DYN, particularly during the stress-induced reinstatement of reward-seeking behavior. However, emerging studies suggest that these peptides exhibit opposing effects, particularly within hypothalamic pathways. During alcohol dependence—especially under acute stress conditions—DYN’s ability to counteract OX-driven drug-seeking behavior may diminish, potentially exacerbating the vulnerability to relapse. To investigate the role of OX, CRF, and DYN signaling in the IL during stress-induced alcohol seeking during alcohol abstinence (~3 weeks), the present study sought to (1) determine whether blocking IL CRF_1_ receptors (with CP154,526), OX_1/2_ receptors (with the dual OX receptor antagonist [DORA] suvorexant [SUV]), and KOPs (with nor-binaltorphimine [norBNI]) attenuates the stress-induced reinstatement of alcohol-seeking behavior, (2) evaluate potential synergistic or opposite interactions between OX_1/2_ receptor, CRF_1_ receptor, and KOP signaling by co-administering these antagonists, and (3) analyze alcohol-dependence-induced changes in *Hcrtr1*, *Hcrtr2*, *Crhr1*, and *Oprk1* mRNA expression in the IL to further clarify receptorlevel mechanisms.

## 2. Methods

### 2.1. Animals

A total of 120 Wistar rats (60 males and 60 females; Charles River Laboratories, Hollister, CA, USA), weighing 150-170 g upon arrival (~6–7 weeks old), were pair-housed in a temperature- and humidity-controlled vivarium under a reverse 12 h/12 h light/dark cycle (lights off at 8:00 a.m.). Food and water were provided *ad libitum*. Rats were acclimated to the housing and handling conditions for 1 week before the experiments. Therefore, the rats began the alcohol self-administration training when they were ~7–8 weeks old, corresponding to end of adolescence/emerging adulthood. All experimental procedures strictly adhered to the National Institutes of Health *Guide for the Care and Use of Laboratory Animals* and Animal Research: Reporting In Vivo Experiments (ARRIVE) Guidelines (National Research Council, 2011; Percie du Sert et al., 2020) and were approved by The Scripps Research Institute’s Institutional Animal Care and Use Committee.

### 2.2. Drugs

The DORA SUV (AdooQ Bioscience, Irvine, CA, USA), the CRF_1_ receptor antagonist CP154,526 (Tocris Bioscience, Bristol, UK), the KOP antagonist norBNI (Abcam, Waltham, MA, USA), and SUV + CP154,526 and SUV + norBNI combinations were dissolved in 100% dimethylsulfoxide (DMSO; Sigma Aldrich, St. Louis, MO, USA). Control vehicle (VEH)-treated animals received 100% DMSO only. Pure DMSO was utilized because of CP154,526 and SUV’s limited solubility in aqueous vehicles at the required concentration.

### 2.3. Alcohol self-administration training

**Fig. 1.**
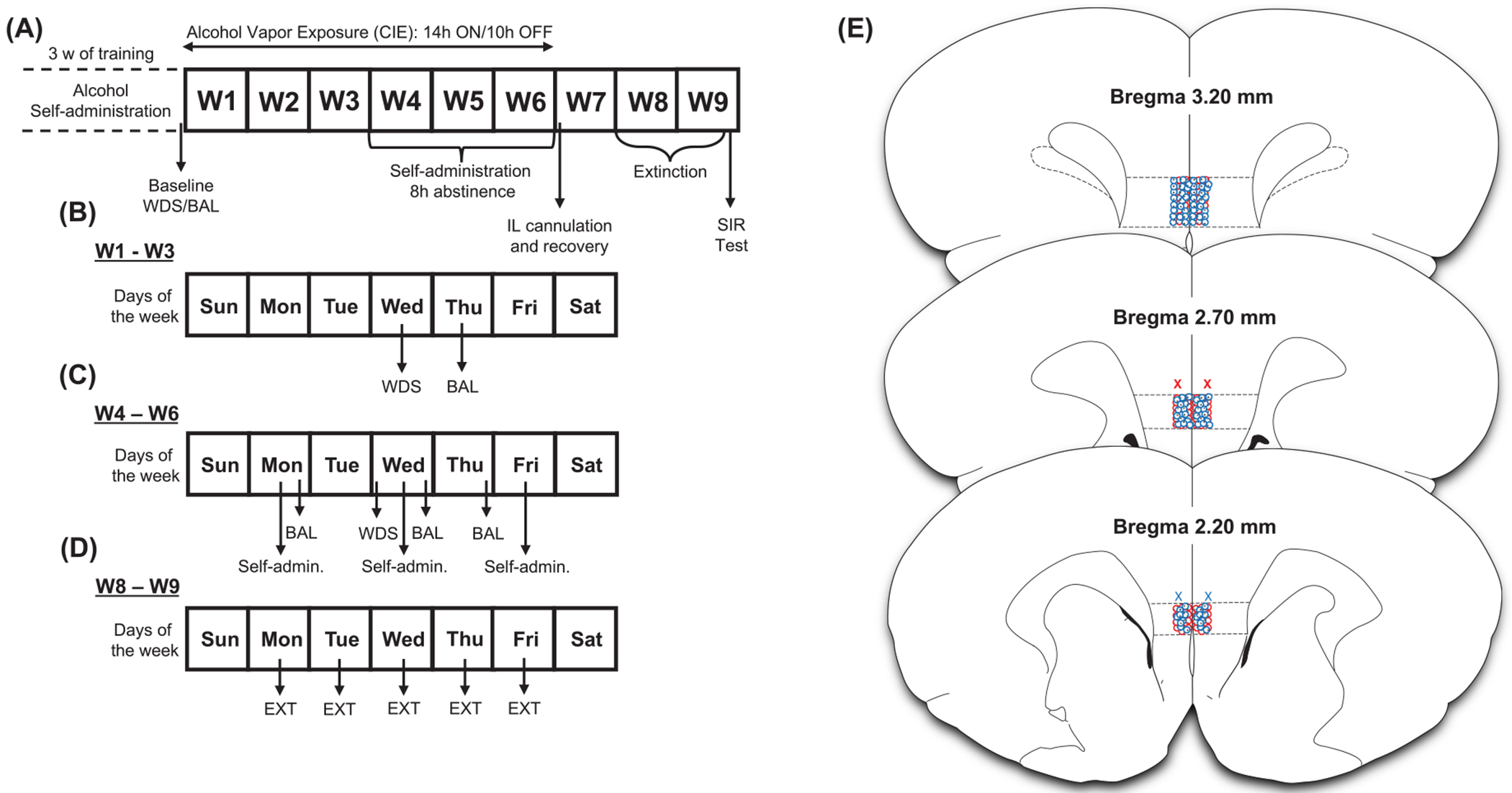
Timeline of the experimental procedures. **(A)** Male and female Wistar rats underwent 21 alcohol self-administration training sessions. Following training completion, baseline somatic withdrawal signs and blood alcohol levels were recorded. **(B)** Between weeks 1 and 3 of chronic intermittent alcohol vapor exposure, rats were scored for somatic withdrawal signs 8 h after the vapor was turned OFF on Wednesday, and blood alcohol levels were recorded 30 min before the alcohol vapors were turned OFF on Thursdays. **(C)** The rats underwent self-administration sessions three times per week (Monday, Wednesday, and Friday) 8 h after the alcohol vapor turned OFF between weeks 4 and 6 of alcohol vapor exposure. **(D)** Following 1 week of recovery from IL cannulation, the rats underwent extinction sessions twice daily. **(E)** Representation of injection sites. o, rats with correct injection sites; × , rats with missed injection sites. Blue o denotes male rats. Red o represents females. BAL, blood alcohol level; IL, infralimbic cortex; WDS, somatic withdrawal signs; W, week. (For interpretation of the references to colour in this figure legend, the reader is referred to the Web version of this article.)

The rats underwent operant alcohol self-administration training as previously described (Flores-Ramirez et al., 2024; Flores-Ramirez et al., 2022; Flores-Ramirez et al., 2023; Matzeu and Martin-Fardon, 2020). Briefly, after a 1-week acclimation period, daily 30-min self-administration sessions were conducted in standard operant chambers (Med Associates, St. Albans, VT, USA) under a fixed-ratio 1 (FR1) schedule for 21 days (Fig. 1A). Pressing the active (right) lever resulted in the delivery of 0.1 ml of 10% alcohol solution along with 0.5-s cue light activation. Pressing the inactive (left) lever was recorded but had no programmed outcomes. No fading procedures (e.g., saccharin/sucrose) were required to initiate voluntary alcohol consumption. Alcohol intake (g/kg) was calculated by normalizing active lever presses to daily body weight. Post-session reservoir checks were performed to confirm that all dispensed alcohol was consumed. Baseline self-administration levels were obtained by averaging the last three self-administration training sessions. After the completion of training, rats with comparable active-lever performance were randomly assigned to stress-induced reinstatement or quantitative polymerase chain reaction (qPCR) experiments.

### 2.4. Chronic intermittent ethanol vapor exposure

Following self-administration training, the rats were divided into dependent (*n* = 60 chronic intermittent ethanol [CIE]-exposed; 30 females and 30 males) and nondependent (*n* = 60 air-exposed; 30 females and 30 males) groups. Dependent rats underwent 6 weeks of daily 14-h alcohol vapor cycles that were interspersed with 10-h vapor OFF periods. Blood alcohol levels (BALs) were maintained at 150-250 mg% and measured weekly using a gas chromatography-headspace blood analyzer (Agilent Technologies, Santa Clara, CA, USA). Baseline BALs were measured immediately after the last alcohol self-administration training session (i.e., Day 21, see Fig. 1A). For 3 weeks, all rats remained undisturbed apart from measuring BALs during the last 30 min of vapor exposure (on Thursday) and scoring somatic signs of withdrawal (at 8 h of abstinence) once weekly (on Wednesday; see Fig. 1A). Withdrawal severity was assessed weekly by an observer who was blind to experimental conditions using a validated scale that scores somatic signs of withdrawal, including measures of ventromedial limb retraction, vocalization (i.e., irritability to touch), tail stiffness, abnormal gait, and body tremors. Each of these behaviors was assigned a score of 0-2, based on severity (0 = no signs, 1 = moderate signs, 2 = severe signs). To confirm dependence and assess withdrawal severity, the sum of the five scores (0-10) was used as a quantitative measure (Flores-Ramirez et al., 2024; Flores-Ramirez et al., 2022; Flores-Ramirez et al., 2023; Macey et al., 1996). Baseline withdrawal scores were measured before the last training session (Day 21, Fig. 1A). During weeks 4-6, all rats (dependent and nondependent) completed tri-weekly (Monday, Wednesday, and Friday) FR1 alcohol self-administration sessions 8 h post-vapor termination when BALs were undetectable (Fig. 1C). For weeks 4-6, self-administration levels during each week were obtained by averaging the number of responses obtained on Monday, Wednesday and Friday of that particular week. To further verify alcohol dependence, BALs were measured immediately after self-administration sessions at weeks 4, 5, and 6 of alcohol vapor exposure. This approach was used because this model of alcohol dependence is well-known to induce motivational and somatic signs of withdrawal (Flores-Ramirez et al., 2024; Flores-Ramirez et al., 2022; Flores-Ramirez et al., 2023; Vendruscolo and Roberts, 2014). Air-exposed rats underwent identical procedures (BAL testing, withdrawal scoring) as dependent subjects.

### 2.5. Infralimbic cortex cannulation

After 6 weeks of alcohol vapor exposure, the rats were removed from the alcohol vapor chambers and started a ~3-week period (Fig. 1A). The rats were implanted with bilateral guide cannulas (22-gauge) that targeted the IL (stereotaxic coordinates: anterior/posterior, +3.2 mm; medial/lateral, ±0.75 mm; dorsal/ventral, −2.6 mm from bregma) (Paxinos and Watson, 1998). Cannulas were positioned 2 mm above the final injection site. After a 7-day surgical recovery period, the rats started extinction training (see Fig. 1A).

### 2.6. Extinction training

Extinction sessions mirrored prior alcohol self-administration sessions, but alcohol delivery was withheld (Fig. 1D). To acclimate the rats to footshock stress, they were placed in operant chambers 15 min before each session. Afterward, levers were extended to initiate the 30-min extinction session. Over 12 sessions (two 30-min sessions per day for 6 days), the rats learned to disassociate active lever pressing from alcohol reward. Twenty-four hours after the last extinction session, the rats underwent a sham microinjection for habituation to intracranial injections, in which injectors were inserted into IL guide cannulas for 2 min (Plastics One, Roanoke, VA, USA). The rats were then returned to their home cages for 2 min before a 15-min operant chamber habituation period. Finally, the levers were subsequently extended into the operant chambers to test the rats under extinction conditions.

### 2.7. Stress-induced reinstatement

Twenty-four hours after the sham injection, the rats received intra-IL microinjections of vehicle (DMSO), SUV (15 μg/0.5 μl/side; (Dong et al., 2015; Flores-Ramirez et al., 2023; Hsiao et al., 2012; Matzeu and Martin-Fardon, 2020), CP154,526 (0.6 μg/0.5 μl/side; (Flores-Ramirez et al., 2022; Hwa et al., 2013), norBNI (4 μg/0.5 μl; (Matzeu et al., 2018; Whitfield et al., 2015), or their combinations (i.e., SUV + CP154,526 or SUV + norBNI) to test their effects on the stress-induced reinstatement of alcohol-seeking behavior. The rationale of using SUV as the DORA of choice was for 3 reasons: (1) it is an FDA-approved DORA marketed by Merck as Belsomra for the treatment of insomnia (Michelson et al., 2014), (2) there is an interest in repurposing it for the treatment of AUD (e.g., Aubin, 2024; Campbell et al., 2020) and (3) because our group found it to be very efficient at blocking the stress-induced reinstatement of alcohol-seeking behavior in post-dependent rats (Flores-Ramirez et al., 2022). Because of norBNI’s documented long-lasting actions (Endoh et al., 1992), animals in these groups were injected 24-h before combination treatment or reinstatement tests. Injections were delivered via a Harvard 22 syringe pump using injectors that extended 2.0 mm beyond the guide cannulas (0.5 μl/min over 1 min). For the drug combinations, both agents were co-administered within the same 0.5-μl injection volume. The injectors remained in place for one additional minute post-infusion to ensure diffusion away from the injector tip. The rats were gently held during the procedure to minimize stress, and they were returned to their home cages for 2 min before being subjected to footshock stress (15 min; variable intermittent electric footshock, 0.5 mA; duration, 0.5 s; mean shock interval, 40 s; (Flores-Ramirez et al., 2022; Flores-Ramirez et al., 2023; Martin-Fardon et al., 2000; Matzeu and Martin-Fardon, 2020; Sidhpura et al., 2010; Zhao et al., 2006). Two minutes post-stress, levers were extended into the operant chamber, and responses were recorded for 30 min. To verify intra-IL injection sites, the rats were deeply anesthetized by CO_2_ inhalation, and their brains were rapidly harvested, snap-frozen in methyl butane, and cut into 40 μm sections using a cryostat (Leica CM3050S, Leica Biosystems Nusslich, Heidelberg, Germany). Using an adult rat brain atlas as a reference (Paxinos and Watson, 1998), the injection sites were verified, and off-target cannulations were excluded from the study (Fig. 1E).

### 2.8. Measures of Hcrtr1, Hcrtr2, Crhr1, and Oprk1 mRNA abundance by qPCR

A separate group of rats (*n* = 16; 8 females and 8 males) for gene expression analysis underwent the same behavioral procedures, including alcohol dependence induction and extinction training, but did not receive IL cannulation or injections or undergo reinstatement testing. The rats were euthanized 24 h after the last extinction session, corresponding to the time when the behavioral group of rats was tested for stress-induced reinstatement. Brains were rapidly harvested, snap-frozen in methylbutane, and stored at −80 °C. An additional control group that was experimentally naive to all conditions (*n* = 8; 4 females and 4 males) was also prepared, and their brains were processed similarly. Brains were dissected into serial coronal sections, and the IL was collected using tissue punches (World Precision Instruments, Sarasota, FL, USA). RNA was isolated (Zymo Research RNA concentrator-5 kits, Irvine, CA, USA), quantified (NanoDrop, 2000c spectrophotometer, Thermo Fisher Scientific, Waltham, MA, USA), and reverse-transcribed to cDNA using 5X mix, iScript, reverse transcription, and Supermix for real-time qPCR (RT-qPCR) with the CFX 384 Real-Time System (BioRad, Hercules, CA, USA). To amplify cDNA, SYBR, iTap Universal SYBR, and Green Supermix were used and analyzed using duplicate samples. Cycle threshold (Ct) values were determined, and changes in gene expression were assessed using the ΔCt method with β-actin as the housekeeping reference gene. The forward and reverse primer sequences of the antisense oligonucleotides were the following: *β-actin* (forward, 5′-ATC TGG CAC ACC TTC-3’; reverse, 5′-AGC CAG GTC CAG ACG CA-3′), *Hcrtr1* (forward, 5′-CCC TCA ACT CCA GTC CTA GC-3’; reverse, 5′-CAG GGA GGG CCT ATA ATT GA-3′), *Hcrtr2* (forward, 5′-CCA TGT TGG GGT GCT TA-3’; reverse, 5′-TCC CCC TCT CAT AAA CTT GG-3′), *Crhr1* (forward, 5′-TGC CAG GAG ATT CTC AAC GAA-3’; reverse, 5′-AAA GCC GAG ATG AGG TTC CAG-3′), and *Oprk1* (forward, 5′-CCA AAG TCA GGG AAG ATG TGG A-3’; reverse, 5′-TCA AGC GCA GGA TCA TCA GG-3′).

### 2.9. Statistical analysis

Self-administration data during chronic alcohol vapor exposure (baseline *vs*. weeks 4-6), withdrawal score values (log10-transformed for the statistical analysis because of the ordinal characteristics of the withdrawal score values, back-transformed for graphical representations), and BALs after self-administration sessions were analyzed using two-way repeated-measures analysis of variance (ANOVA), with time and alcohol dependence as factors. The effect of SUV, CP154,526, norBNI, or their combinations (i.e., SUV + CP154,526 or SUV + norBNI) on active lever presses during the stress-induced reinstatement of alcohol-seeking was analyzed using a two-way ANOVA, with alcohol dependence and treatment as sources of variance. The gene expression data were analyzed using one-way ANOVA. Significant interactions and main effects were followed by the Tukey *post hoc* test for all ANOVAs. The data are expressed as the mean + SEM. Values of *p* < 0.05 were considered statistically significant. The analyses were performed using GraphPad Prism 10.4.2 software.

## 3. Results

Among rats that were designated for the stress-induced reinstatement study, eight were excluded (two never acquired self-administration [1 male and 1 female], two had cannula misplacements [1 male and 1 female], and 5 had post-operative health complications [3 males and 2 females]), reducing the total number of animals to 111 (*n* = 87 for the stress-induced reinstatement experiment, *n* = 16 for the qPCR assay in dependent *vs*. nondependent rats, and *n* = 8 for the qPCR assay in experimentally naive rats).

### 3.1. Alcohol self-administration and escalation

**Fig. 2.**
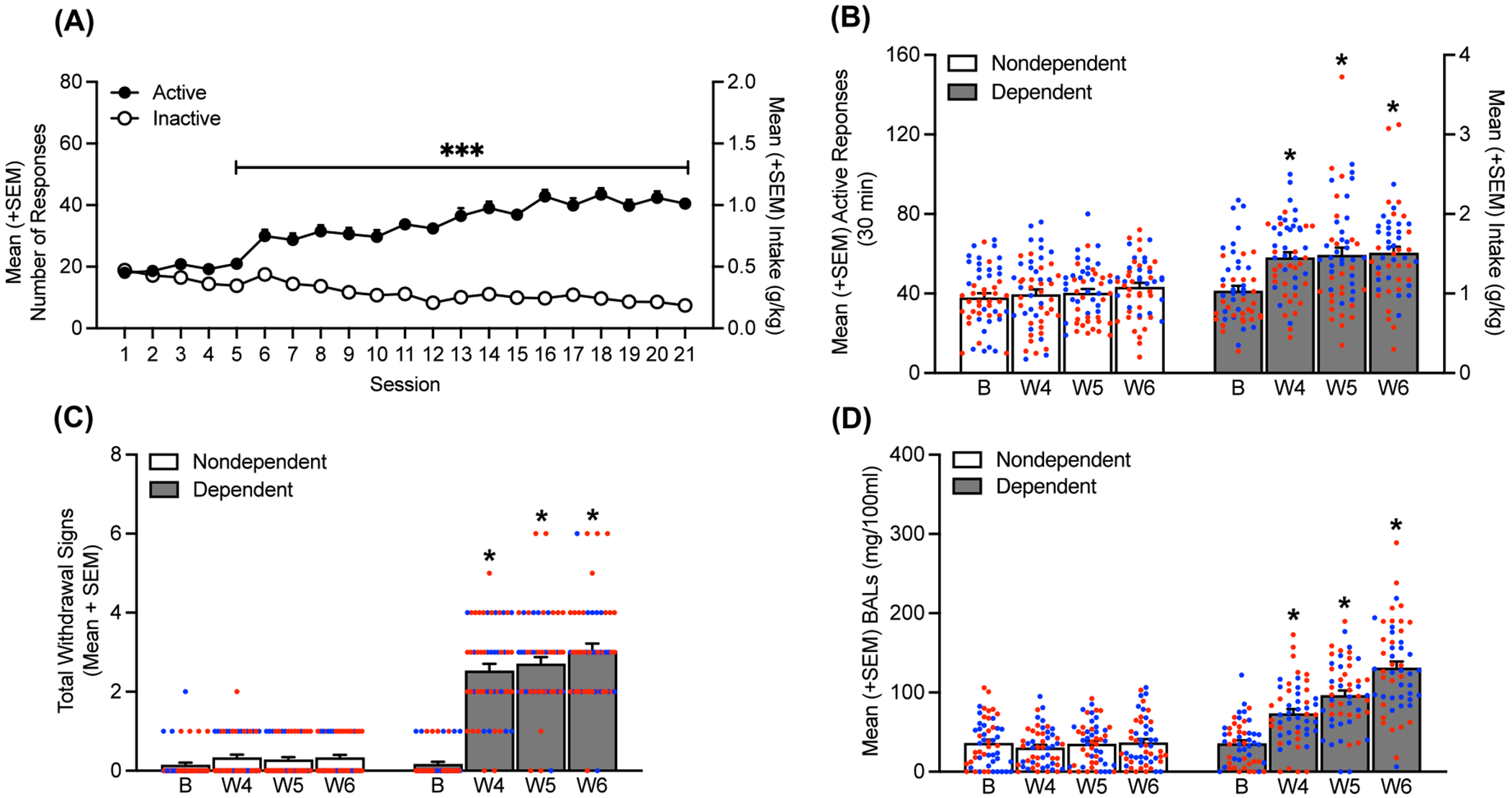
Time course of alcohol self-administration over 21 sessions of training and the escalation of drinking during weeks 4, 5, and 6 of chronic intermittent alcohol vapor exposure. **(A)** Starting in session 5, the rats responded more on the active lever, and stable self-administration began in session 6. **(B)** At weeks 4, 5, and 6 of chronic intermittent alcohol vapor exposure, dependent rats exhibited an increase in active lever responses (and intake). **(C)** During acute abstinence, an increase in somatic withdrawal signs (WDS) was observed in dependent rats at weeks 4, 5, and 6 of chronic intermittent alcohol vapor exposure. **(D)** After the self-administration sessions at weeks 4, 5, and 6 of chronic intermittent alcohol vapor exposure, alcohol-dependent rats had higher blood alcohol levels than nondependent rats. ***p < 0.001, *vs.* inactive lever; *p < 0.05, *vs.* respective baseline. Blue dots denote data from male rats. Red dots denote data from females. B, baseline; BAL, blood alcohol level; W, week. (For interpretation of the references to colour in this figure legend, the reader is referred to the Web version of this article.)

Rats (*n* = 103, including rats that were designated for the mRNA analyses) acquired alcohol self-administration over 21 sessions of training (30 min/day; two-way repeated-measures ANOVA; time: *F*_20,4160_ = 10.84, *p* < 0.05; lever: *F*_1,208_ = 260.98, *p* < 0.05; time × lever interaction: *F*_20,4160_ = 38.74, *p* < 0.05;Fig. 2A). The Tukey *post hoc* test confirmed that the number of responses at the active lever was significantly higher than responses at the inactive lever starting at session 5 (*p* < 0.05; Fig. 2A).

Sex did not significantly contribute to the variance in alcohol intake (three-way ANOVA; Sex: *F*_1,404_ = 57.02, *p* < 0.05; Time: *F*_3,404_ = 5.43, *p* < 0.05; Alcohol dependence: *F*_1,404_ = 104.31, *p* < 0.05; Sex × Time: *F*_3,404_ = 2.16, *p* > 0.05; Sex × alcohol dependence: *F*_1,404_ = 3.68, *p* > 0.05; Time × alcohol dependence: *F*_3,404_ = 8.79, *p* < 0.05; Sex × Time × Alcohol dependence: *F*_3,404_ = 1.73, *p* > 0.05), somatic withdrawal signs (three-way ANOVA; Sex: *F*_1,404_ = 4.82, *p* > 0.05; Time: *F*_3,404_ = 74.01, *p* < 0.05; Alcohol dependence: *F*_1,404_ = 510.29, *p* < 0.05; Sex × Time: *F*_3,404_ = 1.30, *p* > 0.05; Sex × alcohol dependence: *F*_1,404_ = 1.04, *p* > 0.05; Time × alcohol dependence: *F*_3,404_ = 57.18, *p* < 0.05; Sex × Time × Alcohol dependence: *F*_3,404_ = 0.45, *p* > 0.05), or BALs (three-way ANOVA; Sex: *F*_1,404_ = 0.49, *p* > 0.05; Time: *F*_3,404_ = 34.79, *p* < 0.05; Alcohol dependence: *F*_1,404_ = 201.57, *p* < 0.05; Sex × Time: *F*_3,404_ = 0.26, *p* > 0.05; Sex × alcohol dependence: *F*_1,404_ = 1.20, *p* > 0.05; Time × alcohol dependence: *F*_3,404_ = 32.27, *p* < 0.05; Sex × Time × Alcohol dependence: *F*_3,404_ = 2.17, *p* > 0.05); thus, data obtained from both male and female rats were analyzed together. During weeks 4, 5, and 6 of alcohol vapor exposure, alcohol-dependent animals exhibited a significant increase in active lever responses (and intake), a measure that was obtained by averaging the intake data that were recorded on Monday, Wednesday, and Friday of that week (*p* < 0.05, Tukey *post hoc* test *vs.* baseline following two-way repeated-measures ANOVA; time: *F*_3,309_ = 17.14, *p* < 0.05; alcohol dependence: *F*_1,103_ = 26.70, *p* < 0.05; time × alcohol dependence interaction: *F*_3,309_ = 8.20, *p* < 0.05;Fig. 2B). During weeks 4, 5, and 6 of alcohol vapor exposure, alcohol-dependent animals exhibited significantly higher somatic withdrawal signs at an acute abstinence point (8 h after vapors were OFF; *p* < 0.05, Tukey *post hoc* test *vs.* baseline following two-way repeated-measures ANOVA; time: *F*_3,309_ = 98.99, *p* < 0.05; alcohol dependence: *F*_1,103_ = 288.37, *p* < 0.05; time × alcohol dependence interaction: *F*_3,309_ = 76.46, *p* < 0.05;Fig. 2C). Alcohol-dependent animals had higher BALs after self-administration sessions at weeks 4, 5, and 6 (*p* < 0.05, Tukey *post hoc* test *vs.* baseline following two-way repeated-measures ANOVA; time: *F*_3,309_ = 45.25, *p* < 0.05; alcohol dependence: *F*_1,103_ = 115.77, *p* < 0.05; time × alcohol dependence interaction: *F*_3,309_ = 42.20, *p* < 0.05; Fig. 2D).

### 3.2. Extinction and stress-induced reinstatement

**Fig. 3.**
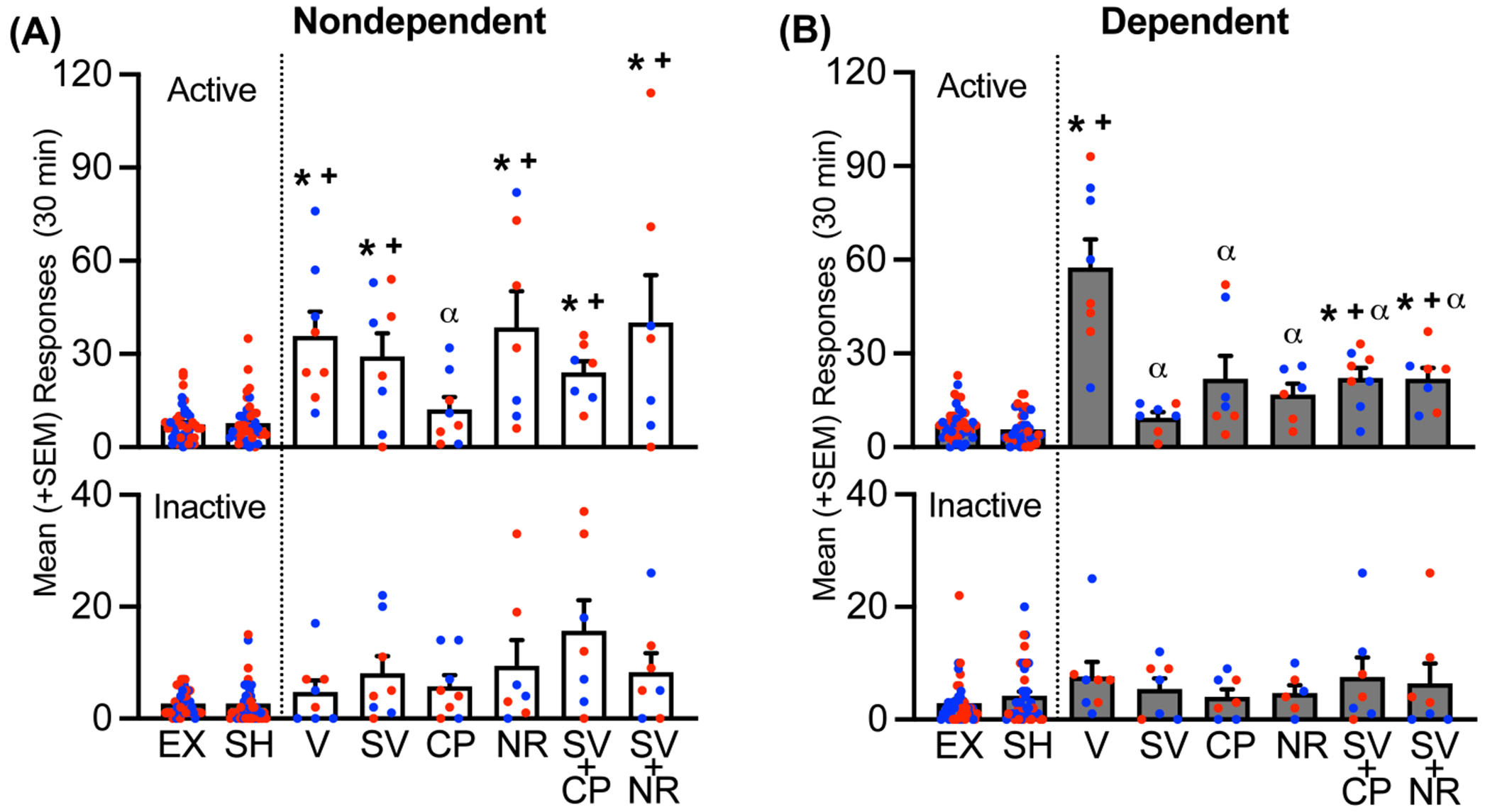
Intermittent footshock stress precipitated alcohol-seeking behavior in rats that received vehicle (VEH) in both dependent and nondependent groups. In nondependent rats, the administration of CP154,526 in the IL prevented the stress-induced reinstatement of alcohol seeking, an effect that was reversed by the co-administration of suvorexant. In dependent rats, suvorexant, CP154,526, norBNI, and the suvorexant + CP154,526 and suvorexant + norBNI combinations decreased the stress-induced reinstatement of alcohol-seeking behavior. The effect of suvorexant was attenuated by the co-administration of CP154,526 or norBNI. No differences in inactive lever responses were observed. *p < 0.05, *vs.* respective EXT; ^+^*p* < 0.05, *vs.* respective sham; ^α^*p* < 0.05, *vs.* respective VEH. Blue dots denote data from males. Red dots denote data from females. EX, extinction; SH, sham; V, vehicle; SV, suvorexant; CP, CP154,526; NR, norBNI. (For interpretation of the references to colour in this figure legend, the reader is referred to the Web version of this article.)

Sex did not significantly contribute to the variance of the model in the stress-induced reinstatement paradigm (three-way ANOVA; Sex: *F*_1,231_ = 1.74, *p* > 0.05; Treatment: *F*_7,231_ = 34.00, *p* < 0.05; Alcohol dependence: *F*_1,231_ = 6.22, *p* < 0.05; Sex × Treatment: *F*_7,231_ = 0.87, *p* > 0.05; Sex × alcohol dependence: *F*_1,248_ = 1.96, *p* > 0.05; Treatment × alcohol dependence: *F*_7,231_ = 6.63, *p* < 0.05; Sex × Treatment × Alcohol dependence: *F*_1,231_ = 1.21, *p* > 0.05); thus, data obtained from both male and female rats were analyzed together. Over 12 sessions of extinction training, the number of responses at the active lever gradually decreased until the total number of responses on the active lever was undistinguishable from the number of responses at the inactive lever, similar to earlier studies (e.g., (Flores-Ramirez et al., 2022; Flores-Ramirez et al., 2023). Following extinction, sham injections did not reinstate or suppress any behavior, and the rat’s performance at the active and inactive levers remained at the level of extinction (Fig. 3A and B).

Stress precipitated the reinstatement of alcohol-seeking behavior in both nondependent and dependent rats under VEH condition (Fig. 3A and B). In nondependent rats, the administration of SUV, norBNI, and the SUV + norBNI combination did not modify the stress-induced reinstatement of alcohol-seeking behavior. In contrast, the administration of CP154,526 prevented stress-induced reinstatement of alcohol-seeking behavior, an effect that was nullified by co-administering SUV (*p* < 0.05, Tukey *post-hoc* tests *vs.* EXT, Sham, and VEH following two-way ANOVA; Treatment: *F*_7,248_ = 33.76, *p* < 0.05; Alcohol dependence: *F*_1,248_ = 4.34, *p* < 0.05; Treatment × alcohol dependence: *F*_7,248_ = 4.45, *p* < 0.05;Fig. 3A). In dependent rats, the administration of SUV, CP154,526, norBNI, as well as the SUV + CP154,526 and SUV + norBNI combinations significantly reduced the stress-induced reinstatement of alcohol-seeking behavior *vs*. VEH (*p* < 0.05, Tukey *post-hoc* tests *vs.* VEH following two-way ANOVA; Treatment: *F*_7,248_ = 33.76, *p* < 0.05; Alcohol dependence: *F*_1,248_ = 4.34, *p* < 0.05; Treatment × alcohol dependence: *F*_7,248_ = 4.45, *p* < 0.05;Fig. 3B) with SUV, CP154,526 and norBNI notably reducing the number of responses to the level of extinction and sham (*p* < 0.05, Tukey *post-hoc* tests *vs.* EXT and Sham following two-way ANOVA). The efficacy of SUV at reducing the stress-induced reinstatement of alcohol-seeking behavior was significantly attenuated by co-administration with CP154,526 and norBNI (*p* < 0.05, Tukey *post-hoc* tests *vs.* EXT and Sham following two-way ANOVA). No differences in inactive lever responses were observed, regardless of a history of alcohol-dependence and treatment condition (*p* > 0.05;Fig. 3A and B).

### 3.3. Measures of Hcrtr1, Hcrtr2, Crhr1, and Oprk1 mRNA abundance by qPCR

**Fig. 4.**
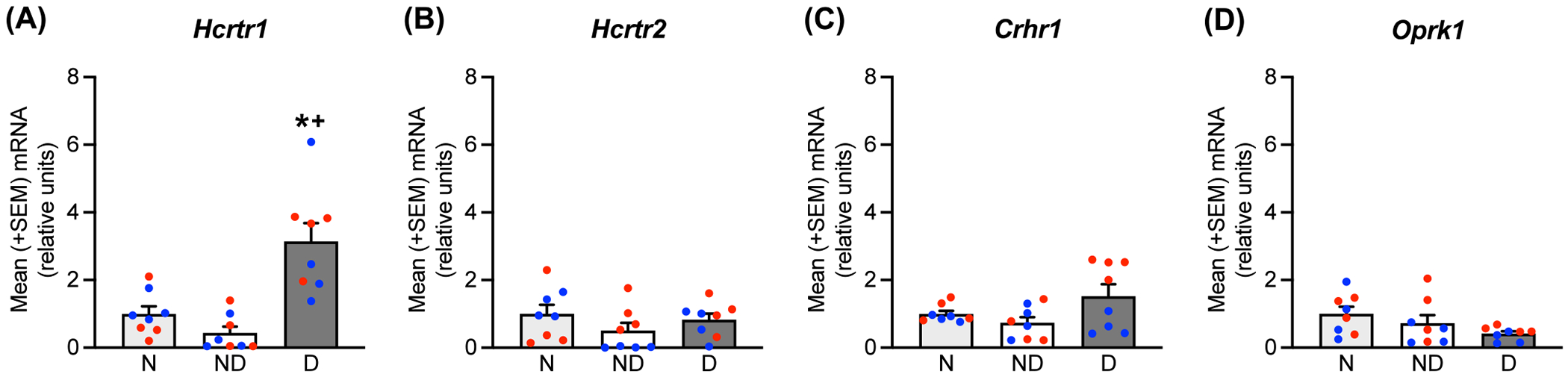
Effects of chronic intermittent alcohol vapor exposure on the mRNA expression of *Hcrtr1*, *Hcrtr2*, *Crhr1*, and *Oprk1* in the IL during abstinence, corresponding to the time when the stress-induced reinstatement tests were conducted. At the time of testing, increases in *Hcrtr1* **(A)** mRNA expression in the IL were found, whereas no significant changes in *Hcrtr2* **(B)** *Crhr1* (**C)** or *Oprk1* **(D)** were observed. *p < 0.05, *vs.* nondependent animals, ^+^*p* < 0.05, *vs.* naive animals. Blue dots denote data from males. Red dots denote data from females. N, naive; ND, nondependent; D, dependent. (For interpretation of the references to colour in this figure legend, the reader is referred to the Web version of this article.)

Analyses of mRNA expression in the IL showed that alcohol dependence significantly increased *Hcrtr1* (Tukey post hoc tests, *p* < 0.05, vs. naive, and *p* < 0.05, vs. nondependent following a one-way ANOVA: *F*_2,21_ = 16.14, *p* < 0.05). No changes in *Hcrtr2*, *Crhr1*, or *Oprk1* mRNA expression were observed, regardless of experimental condition (*p* > 0.05, respectively;Fig. 4B–D).

## 4. Discussion

The present study investigated the role of OX, CRF, and DYN transmission in the IL and OX/CRF and OX/DYN interactions during the stress-induced reinstatement of alcohol-seeking behavior in dependent rats, alongside molecular changes in *Hcrtr1*, *Hcrtr2*, *Crhr1*, and *Oprk1* mRNA expression in the IL during abstinence. Importantly, alcohol-dependent rats exhibited the escalation of self-administration during chronic vapor exposure, aligning with prior findings (Flores-Ramirez et al., 2024; Flores-Ramirez et al., 2022; Flores-Ramirez et al., 2023; Matzeu et al., 2018; Matzeu and Martin-Fardon, 2020; O’Dell et al., 2004). Consistent with previous studies (e.g., Flores-Ramirez et al., 2024; Flores-Ramirez et al., 2022; Flores-Ramirez et al., 2023; Le et al., 1999; Martin-Fardon et al., 2000), the present findings confirm that intermittent, unpredictable footshock stress reinstated alcohol-seeking behavior. In nondependent rats, intra-IL administration of the CRF1 receptor antagonist CP154,526 blocked reinstatement, an effect that was reversed by the co-administration of the DORA SUV, mirroring earlier observations (Flores-Ramirez et al., 2022). In dependent rats, the blockade of OX_1/2_ receptor signaling, CRF_1_ receptor signaling, and KOP signaling in the IL prevented the stress-induced reinstatement of alcohol-seeking behavior. Notably, the effect of SUV was significantly attenuated by the co-administration of CP154,526 or norBNI. Finally, alterations of *Hcrtr1* mRNA gene expression were apparent following extinction, suggesting a lasting change in OX_1_ receptor signaling. These findings highlight the dysregulation of IL OX/CRF/DYN transmission by alcohol dependence and functional interactions between their transmission during the stress-induced reinstatement of alcohol-seeking behavior.

The blockade of OX_1/2_ receptors in the IL with SUV selectively prevented the stress-induced reinstatement of alcohol-seeking behavior in alcohol-dependent rats. This is consistent with previous studies that showed that the pharmacological blockade of OX receptors selectively reduced alcohol-related behaviors without affecting behavior directed at non-alcohol rewards (i.e., sweet solutions). For example, OX_1_ receptor blockade decreased progressive-ratio alcohol responding in male inbred alcohol-preferring (iP) rats (Jupp et al., 2011) and suppressed alcohol but not SuperSac (i.e., a palatable sweet solution) seeking in male Wistar rats (Martin-Fardon and Weiss, 2014). Similarly, the subcutaneous administration of JNJ-10397049, an OX_2_ receptor antagonist, reduced alcohol but not saccharin intake, an alternative non-alcohol reinforcer, in male Wistar rats (Shoblock et al., 2011). Furthermore, administration of the selective OX_2_ receptor antagonist TCSOX229 in the nucleus accumbens core but not shell decreased alcohol self-administration but not the conditioned reinstatement of alcohol-seeking behavior in male iP rats (Brown et al., 2013). The selectivity of SUV in the present study may stem from the dependence-specific upregulation of OX receptor signaling in the IL. Previous studies found that OX receptor antagonists more effectively reduced alcohol-seeking in dependent or high-alcohol-preferring rats (Flores-Ramirez et al., 2022; Flores-Ramirez et al., 2023; Matzeu and Martin-Fardon, 2020; Moorman and Aston-Jones, 2009; Moorman et al., 2017). These findings suggest that IL OX transmission may become critical only when salience of the reward increases and when anti-reward systems are engaged during withdrawal, driving alcohol motivation via negative reinforcement mechanisms. Notably, similar state-dependent effects of OX receptor blockade have been reported across multiple drug classes. For instance, OX_1/2_ receptor antagonism preferentially reduced cocaine seeking in animals with a history of extended access or high motivation (James et al., 2019; James et al., 2019), and comparable findings have been observed for fentanyl (Fragale et al., 2021; Fragale et al., 2019) and remifentanil (Mohammadkhani et al., 2019). Together, these studies suggest that chronic drug exposure and the resulting escalation of drug taking may shift behavioral control toward OX-dependent circuitry. The results of the present study extend this concept to the IL, indicating that alcohol dependence enhances reliance on OX signaling with this region during stress-induced reinstatement. More broadly, these convergent findings support the notion that heightened motivational states, whether driven by dependence, extended access, or high effort requirements, engage OX pathways to a greater degree, rendering them more sensitive to pharmacological manipulations.

Intra-IL administration of the CRF_1_ receptor antagonist CP154,526 significantly reduced the stress-induced reinstatement of alcohol-seeking behavior in both nondependent and dependent rats, implicating IL CRF_1_ receptor signaling in the mediation of mechanisms that mediate stress-induced relapse. This aligns with prior systemic studies that found that intraperitoneal CP154,526 administration attenuated footshock-induced alcohol-seeking behavior in rats (Le et al., 2000). CRF_1_ receptor antagonists consistently reduce alcohol intake, particularly in heightened motivational states. For example, subcutaneous administration of the CRF_1_ antagonist MPZP significantly reduced dependence-driven increases in alcohol consumption in alcohol-preferring (P) rats *vs*. nondependent P rats (Gilpin et al., 2008). Similarly, systemic antalarmin (another CRF_1_ antagonist) administration decreased alcohol consumption in alcohol-preferring Fawn-Hooded rats that were reared in isolation (Lodge and Lawrence, 2003). Furthermore, the CRF_1_ receptor antagonists D-Phe-CRF_12-41_, CRA1000, CP154,526, and LWH-63 reduced voluntary alcohol drinking via drinking tubes in the home cage and operant alcohol self-administration selectively in dependent male rodents across multiple species and strains (e.g., C57BL/6J mice, Wistar rats, P rats, and Sardinian alcohol-preferring rats) when administered systemically or into the central nucleus of the amygdala (Finn et al., 2007; Funk, O’Dell, Crawford and Koob, 2006; Overstreet et al., 2007; Sabino et al., 2006). More recently, our group reported that intra-IL CP154,526 administration prevented the footshock stress-induced reinstatement of alcohol-seeking behavior in nondependent male Wistar rats (Flores-Ramirez et al., 2022). Altogether, these findings indicate that stress-driven alcohol--seeking behavior is mediated at least partially by CRF_1_ receptor signaling in the IL.

The behavioral findings in the present study are consistent with previous studies that showed that KOP blockade effectively reduced alcohol taking and seeking in subjects with higher motivation for alcohol seeking (i.e., alcohol preference or dependence induction). Indeed, similar to the present study, the extant literature shows that KOP antagonist administration has no effect on basal, non-escalated alcohol consumption but attenuates escalated alcohol intake in post-dependent subjects (Domi et al., 2018; Flores-Ramirez et al., 2024; Walker and Koob, 2008; Walker et al., 2011). For example, in alcohol-dependent male Wistar rats, both the systemic and intracerebroventricular administration of norBNI attenuated withdrawal-induced increases in drinking (Walker and Koob, 2008; Walker et al., 2011), and the oral administration of LY2444296, a short acting KOP antagonist, selectively decreased drinking in male and female alcohol-dependent rats (Flores-Ramirez et al., 2024). Furthermore, in P rats, the oral administration of CERC-501 reduced alcohol intake under free-access conditions (Rorick-Kehn et al., 2014). Thus, KOP dysregulation, via repeated cycles of alcohol use, may theoretically increase stress and dysphoria to elicit the reinstatement of drug-seeking behavior (Wee and Koob, 2010). Indeed, a long-standing theory suggests drug-induced upregulation of the DYN/KOP system underlies negative affective states that are associated with drug withdrawal that likely promote drug seeking well into abstinence (Koob, 2008). Consequently, in animals that are highly motivated to consume alcohol, KOP signaling may be compromised and increase the incentive value of alcohol via negative reinforcement mechanisms.

In nondependent rats, the effects of CP154,526 were prevented by the co-administration of SUV, replicating a previous finding from our laboratory (Flores-Ramirez et al., 2022), whereas in dependent rats, the effect of SUV was prevented by the co-administration of CP154,526, thus revealing an OX_1/2_-CRF_1_ receptor interaction. Interestingly, these effects suggest that the role of OX in determining drug-seeking behavior may be enhanced during stress or when levels of arousal are high (Berridge et al., 2010). This is unsurprising because neuroanatomical evidence supports this interplay. Hypothalamic projections innervate the IL (Date et al., 1999), which expresses OX receptors (Marcus et al., 2001), whereas CRF receptors are also present in the HYP (Winsky-Sommerer et al., 2004), which receives IL inputs (Vertes, 2004), suggesting a reciprocal regulatory loop. Earlier studies reported that systemic OX_1_ receptor antagonism blocked the stress (yohimbine)-induced reinstatement of alcohol-seeking behavior without altering locomotion, implicating OX-CRF crosstalk (Richards et al., 2008). Furthermore, OX cells display greater activation during yohimbine-induced the reinstatement of alcohol-seeking behavior (Kastman et al., 2016; Richards et al., 2008), and blocking OX receptor activity attenuated the footshock stress-induced reinstatement of alcohol-seeking behavior in both male and female Wistar rats (Flores-Ramirez et al., 2022; Flores-Ramirez et al., 2023; Matzeu and Martin-Fardon, 2020). Altogether, these findings demonstrate a functional, albeit complex, OX_1/2_-CRF_1_ receptor interplay in the IL during stress-driven alcohol seeking.

Another major finding of the present study was that norBNI prevented some of SUV’s effects in dependent rats, suggesting of an interaction between OX_1/2_ receptor and KOP signaling in the IL during the footshock stress-induced reinstatement of alcohol-seeking behavior. Previous research demonstrated that OX_1_ receptor antagonism (via systemic or intra-VTA administration of SB334867) elevated intracranial self-stimulation thresholds in mice, an effect that was blocked by pretreatment with norBNI, revealing an interaction between OX_1/2_ receptor and KOP signaling in the VTA. In mouse VTA slices, 65.4% of dopaminergic neurons responded to both OX and DYN with no net change in firing, whereas certain subsets preferentially responded to OX (16.9%) or DYN (7.7%) (Muschamp et al., 2014). In contrast, exogenous OX and DYN application in the lateral HYP exerted opposing effects on neuronal firing (Li & van den Pol, 2006). Furthermore, the optical stimulation of lateral HYP OX/DYN neurons modulated VTA dopamine cell firing, in which OX_1_ receptor antagonism blocked excitation and KOP antagonism blocked inhibition, suggesting that peptide release into the VTA mediates these changes (Mohammadkhani et al., 2024). Another study also found that OX administration in the PVT reinstated cocaine-seeking behavior in male cocaine-experienced Wistar rats, whereas co-administering DYN suppressed the OX-induced reinstatement of cocaine-seeking behavior by attenuating OX-driven glutamate release. Importantly, the co-administration of DYN in the PVT with OX did not reduce OX-induced motivation toward a palatable sweet solution, suggesting that drug dependence alters the interaction of OX/DYN transmission in the PVT, potentially reflecting neuroadaptations that are linked to excessive drug use (Matzeu et al., 2018). Altogether, these findings confirm a functional interaction between OX and DYN transmission in the IL that may depend on synaptic peptide levels, receptor distribution, intracellular signaling, and brain region-specific dynamics.

In is important to note that the doses of SUV, CP154,526, norBNI were carefully selected based on prior studies that demonstrated reliable receptor engagement at concentrations that minimize nonspecific behavioral effects (Dong et al., 2015; Flores-Ramirez et al., 2025; Hsiao et al., 2012; Hwa et al., 2013; Illenberger et al., 2024; Matzeu et al., 2018; Whitfield et al., 2015). Therefore, the doses selected here fall within the range previously shown to block OX_1/2_, CRF1, and KOP receptor signaling and resulting in the attenuation of alcohol-seeking behavior without altering general locomotion, allowing to confidently state that the behavioral changes observed in the present investigation are attributed to pharmacological specificity rather than general nonspecific behavioral impairment. Although the doses selected produced only a partial attenuation of SUV’s efficacy in dependent rats, it remains possible that higher doses of either antagonist could have yielded a more complete reversal. Nevertheless, increasing the dose would also increase the likelihood of off-target actions, including interference with other neuromodulatory system, which would complicate the interpretations of the effects on alcohol-seeking behavior. As such, the selected doses reflect a balance between achieving a meaningful receptor blockade while maintaining behavioral specificity, which is consistent with established intracranial pharmacological approaches.

In rats with a history of alcohol dependence, blocking OX_1/2_ and CRF1 receptors, individually, within the IL prevented the stress-induced reinstatement of alcohol-seeking behavior, whereas concomitant blockade modulated that effect. A similar non-additive pattern was observed after OX_1/2_ receptor and KOP antagonism. Though speculative, these findings may be most parsimoniously explained by pathway interactions within an integrated neuromodulatory hub in the IL. OX_1/2_, CRF_1_ and KOP receptors engage overlapping intracellular transducers (i. e., G proteins, beta-arrestins, and mitogen-activated protein kinase cascades) and can form physical and functional complexes such that antagonism of single receptor disrupts a pro-reinstatement signaling arm and reduces circuit excitability, while simultaneous antagonism can reconfigure the complex to favor alternative G-protein or arrestin pathways that restore downstream signaling sufficient for reinstatement (Koob, 1999; Sakurai et al., 1998; Toneatti et al., 2020; Ward et al., 2013). Concomitant blockade may also alter receptor trafficking and surface expression so that an antagonist that promotes internalization of one promoter when given alone fails to do so when both partners are blocked, thereby preserving partner-receptor signaling and reinstating network activity (Brust et al., 2016; Dale et al., 2022; Smith and Milligan, 2010; Ward et al., 2013). Finally, because OX/CRF/DYN systems regulate local glutamate/γ-amino butyric acid balance and neuropeptide release in the mPFC, combined antagonism may remove opposing inhibitory constraints or trigger compensatory peptide release that produces net disinhibition or IL output neurons and restores the firing patterns that permit reinstatement (Chavkin and Koob, 2016; de Lecea et al., 1998; Haass-Koffler and Bartlett, 2012). Indeed, these speculative molecular, trafficking, and synaptic mechanism may explain the observed non-linear behavioral outcomes and may suggest pathway-selective or biased-ligand approaches when targeting interacting neuromodulatory systems in models of alcohol dependence.

During abstinence (~3 weeks) at the time when the stress-induced reinstatement tests were conducted, an increase in *Hcrtr1* mRNA expression in the IL was measured. These results are consistent with previous studies from our laboratory, in which alcohol-dependent rats during acute (i.e., 8 h) abstinence exhibited increases in *Hcrtr1* and *Hcrtr2* mRNA expression in the PVT (Matzeu and Martin-Fardon, 2020) and IL (Flores-Ramirez et al., 2023). The present results suggest, but do not confirm, that chronic alcohol exposure may dysregulate OX transmission well into late abstinence, leading to possibly heightened IL OX_1_ receptor expression and potentially contributing to dependence-related behaviors (e.g., escalated drinking, relapse vulnerability). Although alcohol dependence was associated with an increase in *Hcrtr1* mRNA expression in the IL, this transcriptional change does not necessarily indicate enhanced OX receptor signaling. Notably, qPCR primarily detects mRNA in cell bodies rather than terminals, and mRNA abundance provides an index of potential receptor availability, but it does not establish changes in receptor protein levels, trafficking, or downstream functional coupling (Lee et al., 2003; Mehra et al., 2003), which limits the generalizability of the results of the present study. Assessing changes in mRNA in rats that are concomitantly exposed to dependence, stress, and pharmacological treatment would certainly be an interesting line of investigation. However, the goal for our approach was to obtain a clearer picture of molecular changes that are induced specifically by alcohol dependence itself and set the stage for the compounds’ ability to prevent reinstatement. Thus, future studies that measure actual protein levels in dependent rats that undergo the stress-induced reinstatement and that are administered pharmacological treatment are warranted.

Presently, the cellular phenotype of IL neurons that may upregulate the expression of *Hcrtr1* mRNA in the context of alcohol dependence remains unknown. Indeed, anatomical and molecular studies in cortex and limbic regions indicate that OX receptors are expressed on both glutamatergic pyramidal neurons and GABAergic interneurons and multiplex RNAscope analyses in the dorsal hippocampus and medial prefrontal cortex revealed that *Hcrtr1* and *Hcrtr2* mRNA are present in both CaMKIIα-positive principal neurons and GAD67/parvalbumin-positive interneurons, with subregion-specific biases in OX_1_ and OX_2_ expression that likely translate into distinct circuit-level effects of OX signaling (Jaszberenyi et al., 2024; Krause et al., 2024). However, comparable cell-type-specific mapping has not yet been performed in the IL after chronic alcohol exposure, and it is unknown whether dependence alters the proportion of OX_1_ receptor-*vs.* OX_2_ receptor-expressing neurons, recruits new neuronal subpopulations to OX modulation, or changes receptor expression within defined microcircuits (e.g., IL projections to the HYP). In this context, future work that combines alcohol-dependence models with high-resolution approaches will be necessary to determine whether alcohol dependence selectively increases the number of OX_1_ receptor-expressing neurons, alters the relative abundance of OX_1_ receptor-*vs*. OX_2_ receptor-expressing cells, or shifts OX receptor expression within specific IL microcircuits that regulate stress-induced alcohol seeking.

Even though the mechanisms at the molecular level remain to be determined, the present findings support, nevertheless, the notion of a functional interaction between OX and CRF and between OX and DYN transmission in the IL and particularly highlight the participation of these interactions in the reinstatement of exacerbated alcohol-seeking behavior following alcohol dependence as a function of heightened stress. These results suggest that disruptions of IL function that are caused by chronic alcohol exposure, particularly maladaptive changes in IL OX, CRF, and DYN transmissions, underly the transition from controlled to compulsive drinking, emphasizing the need for targeted interventions that could restore IL integrity and functionality. The present data underscore the importance of considering multiple pharmacological targets when developing therapies to prevent stress-induced craving and relapse to alcohol use.

## Data Availability

The data that support the findings of this study are available from the corresponding author upon reasonable request. Some data may not be made available because of privacy or ethical restrictions.
